# Superficial sedimentary stocks and sources of carbon and nitrogen in coastal vegetated assemblages along a flow gradient

**DOI:** 10.1038/s41598-018-37031-6

**Published:** 2019-01-24

**Authors:** Rui Santos, Natalia Duque-Núñez, Carmen B. de los Santos, Márcio Martins, A. Rita Carrasco, Cristina Veiga-Pires

**Affiliations:** 1 Center of Marine Sciences (CCMAR), University of Algarve, Faro, Portugal; 2 Centre for Marine and Environmental Research (CIMA), University of Algarve, Faro, Portugal

## Abstract

Coastal vegetated ecosystems are major organic carbon (OC) and total nitrogen (TN) sinks, but the mechanisms that regulate their spatial variability need to be better understood. Here we assessed how superficial sedimentary OC and TN within intertidal vegetated assemblages (saltmarsh and seagrass) vary along a flow gradient, which is a major driver of sediment grain size, and thus of organic matter (OM) content. A significant relationship between flow current velocity and OC and TN stocks in the seagrass was found, but not in the saltmarsh. OC and TN stocks of the saltmarsh were larger than the seagrass, even though that habitat experiences shorter hydroperiods. Mixing models revealed that OM sources also varied along the flow gradient within the seagrass, but not in the saltmarsh, showing increasing contributions of microphytobenthos (17–32%) and decreasing contributions of POM (45–35%). As well, OM sources varied vertically as microphytobenthos contribution was highest at the higher intertidal saltmarsh (48%), but not POM (39%). Macroalgae, seagrass and saltmarsh showed low contributions. Local trade-offs between flow current velocities, hydroperiod and structural complexity of vegetation must be considered, at both horizontal and vertical (elevation) spatial dimensions, for better estimates of blue carbon and nitrogen in coastal ecosystems.

## Introduction

Blue carbon designates the carbon stored and sequestered in marine ecosystems, particularly vegetated coastal systems including seagrasses and saltmarshes^1^. These ecosystems are of global importance for sequestering atmospheric carbon dioxide^2,3^, yet the global decline of seagrasses^4^ and saltmarshes^5^ is not only reducing this important natural carbon sink but may cause the release of the captured carbon dioxide back to the atmosphere^6–11^. For these reasons, the protection and restoration of coastal vegetated ecosystems have been recognized as key global strategies in climate change mitigation^12^. The blue carbon field has grown rapidly in the last years with the aim of getting better estimations of carbon stocks in coastal vegetated ecosystems and their role in the ocean carbon budget^13,14^. However, the understanding of the mechanisms regulating the spatial variability of the organic carbon stocks in costal vegetated ecosystems remains limited^15^.

Organic carbon stocks among seagrass ecosystems vary 18-fold among species^16^. This large variation alerted scientists about the necessity of incorporating species variability into regional and global estimates of seagrass carbon stocks^17^. Surprisingly, despite the exponentially increasing number of studies on seagrass blue carbon stocks, there are still species virtually unrepresented, especially small and fast-growing species. Saltmarshes also store large amounts of carbon, which vary widely with the species, tidal range and intertidal elevation^18^. The hydroperiod and flow dynamics are main drivers of the carbon accumulation along the intertidal range in saltmarshes, even though there is no clear-cut pattern from low to high marsh^19^. Despite commonly co-occurring with seagrasses in temperate regions, the blue carbon stocks of saltmarshes are not being studied at the same pace as of seagrass meadows^18^. Equally interesting is investigating the links among the organic carbon stocks of different communities within coastal vegetated assemblages, since organic matter may be transferred among them or exported to unvegetated adjacent areas^20–22^.

The identification of key environmental drivers of the sedimentary carbon stocks that account for the observed variability is another priority area of research in the blue carbon field^23^. Many recent studies across a wide range of seagrass bioregions and species have reported that the carbon stocks in near-surface sediments are determined by environmental and biological variables^16,17,24–28^. In particular, sediment properties such as high proportion of fine grain size, high porosity and low density are strongly related to high carbon content^17,24^. Part of the variability in these sediment properties may be attributed to the effects of flow current velocity, which is a main driver of sediment grain size, sorting and transport^29^. The efficiency of seagrasses and saltmarshes to filter small particles out of the water column^30^ and to prevent their re-suspension^31^, depend on the hydrodynamics or flow regimes^32,33^. Flow regimes may not only influence the depositional environment of allochthonous organic matter, but also the sources of fresh organic matter, which can be investigated using geochemical properties of the sediment such as stable isotope ratios. Despite the good reasons to consider flow current velocity as a key driver of sedimentary carbon storage by coastal vegetated ecosystems, as highlighted in a recent conceptual model to explain carbon storage in seagrasses^15^, this relationship has been poorly addressed.

Even though coastal vegetated ecosystems are relevant carbon sinks at global scale contributing to climate change mitigation, these ecosystems may be also relevant in other major global biogeochemical cycles. For example, coastal vegetated ecosystems such as saltmarshes and seagrass meadows play a key role in removing excess anthropogenic nitrogen loads in coastal areas^34^, preventing serious and well documented negative environmental impact^35^. Much less information on the sedimentary nitrogen stocks in coastal vegetated areas is available compared to carbon stocks, even though a few recent studies report stocks of both elements^36–38^. Assessing the role of coastal vegetated areas in nitrogen removal by burial is also important in the perspective of the valuation of the ecosystem services, since the market price for nitrogen removal is generally higher than for carbon^39^.

The aim of this work is to assess the sediment surface stocks of organic carbon (OC) and total nitrogen (TN) within the intertidal vegetation of Ria Formosa, a warm-temperate, mesotidal coastal lagoon of southern Portugal. Because relevant gradients of flow current velocity occur within the intricate channel system of the lagoon, we investigated how the sediment grain size, OC and TN, and associated sediment properties, vary along a flow gradient in a tidal channel where extensive meadows of the high intertidal saltmarsh *Spartina maritima* and intertidal seagrass *Zostera noltei* co-occur. Furthermore, we assessed the relative contribution of autochthonous and allochthonous sources to the organic matter pool of each habitat, and if that contribution varied along the flow gradient. The flow gradient herein reported refers to the depth-average current velocities along sample sites, predicted by the application of a numerical model recently developed for Ria Formosa lagoon^40^.

## Results

### Flow gradient and sediment properties

**Figure 1:**
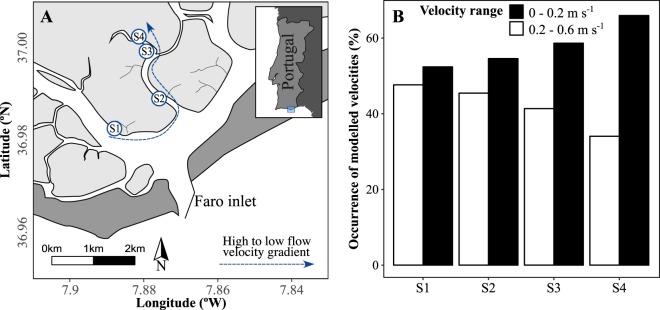
Location of the sampling stations in the Ria Formosa lagoon (South Portugal), and relative frequencies (%) of low (≤0.2 m s^−1^) and high (0.2–0.6 m s^−1^) velocity ranges along the sampling stations.

**Figure 2:**
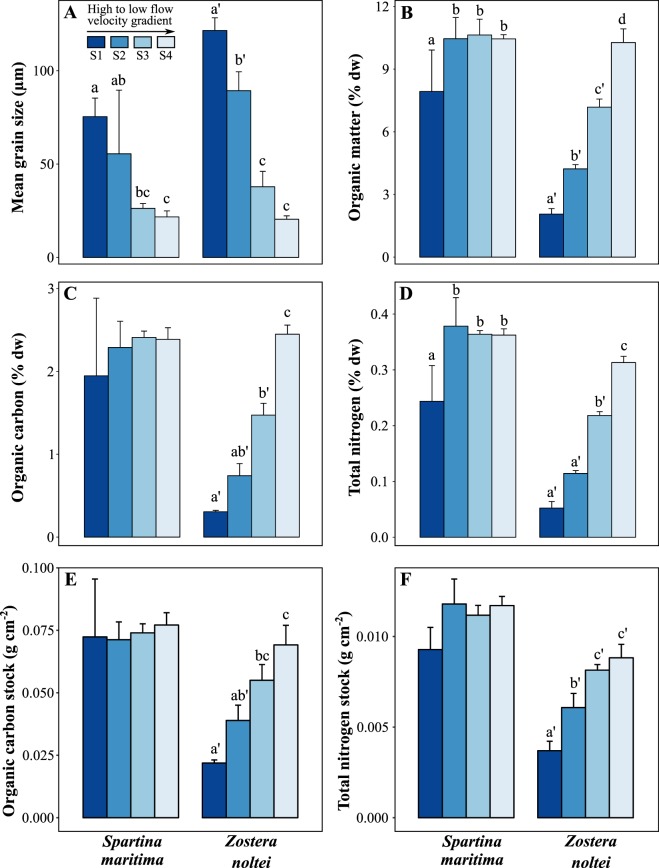
Habitat-specific sediment properties: mean grain size (**A**), percentage of organic matter (**B**), percentage of organic carbon (**C**) percentage of total nitrogen (**D**), organic carbon stock (**E**) and total nitrogen stock (**F**), along the flow gradient, from stations S1 to S4. Bars show means and standard deviation (n = 4). Superscript lettering on bars of each habitat represent post-hoc Tukey pairwise grouping indicating differences among stations within each habitat type, while the symbol ‘ represents differences between habitats for that station.

Predicted depth-averaged current velocities showed a general decrease in the velocity magnitude along the sampling stations (Fig. 1). The relative frequency of lower flow current velocities below or equal to 0.2 m s^−1^ increased from S1 to S4 sampling stations, whereas the relative frequency of higher flow current velocities from 0.2 to 0.6 s^−1^ decreased from S1 to S4. This indicates that conditions for the settlement of fine grain sizes increase from S1 to S4 as opposed to resuspension conditions. The mean grain size of sediments also decreased along the channel, from S1 to S4 in both *S. maritima* and *Z. noltei* habitats (Fig. 2), although this trend was more pronounced for the seagrass. *Z. noltei*, which showed on average higher mean grain size (67.3 ± 42.1 µm, n = 16) than *S. maritima* (44.7 ± 27.8 µm, n = 16) (Fig. 2A, Table 1). The clay content in the sediments showed the same pattern of variation of mean grain size. The percentage of clay mineral (after organic matter removal) varied from 11% in S1 to 22% in S4 in *S. maritima* and from 4% to 22% in *Z. noltei*. The Pearson’s correlation between the clay content and the percentage of organic matter was higher in *Z. noltei* (r = 0.78) than in *S. maritima* (r = 0.59).

**Table 1:** Summary of two-way ANOVA models for sediment properties using habitat (2 levels: seagrass *Zostera noltei* and saltmarsh *Spartina maritima*) and stations (4 levels: from S1 to S4, i.e. from high to low flow current velocity) as fixed factors.

Source of variation	d.f.	MS	F	p
**Organic carbon (% dw)**				
Station	3	2.472	18.69	<0.001
Habitat	1	8.260	62.48	<0.001
Station × Habitat	3	1.227	9.28	<0.001
Residuals	24	0.132		
**Total nitrogen (% dw)**				
Station	3	0.052	57.98	<0.001
Habitat	1	0.211	233.82	<0.001
Station × Habitat	3	0.016	17.86	<0.001
Residuals	24	0.001		
**Organic matter (% dw)**				
Station	3	42.18	53.85	<0.001
Habitat	1	123.72	157.97	<0.001
Station × Habitat	3	15.75	19.89	<0.001
Residuals	24	0.78		
**Grain size (mm)**				
Station	3	10309	55.11	<0.001
Habitat	1	4070	21.76	<0.001
Station × Habitat	3	914	4.88	<0.01
Residuals	24	187		
**Organic carbon stock (g cm** ^**−2**^ **)**				
Station	3	0.0010	10.69	<0.001
Habitat	1	0.0060	63.02	<0.001
Station × Habitat	3	0.0007	7.02	<0.01
Residuals	24	0.0001		
**Total nitrogen stock (g cm** ^**−2**^ **)**				
Station	3	2.2 10^−5^	32.19	<0.001
Habitat	1	1.5 10^−5^	216.97	<0.001
Station × Habitat	3	4.9 10^−5^	7.09	<0.01
Residuals	24	6.8 10^−5^		

A strong spatial variation of the sedimentary organic matter (OM), organic carbon (OC) and total nitrogen (TN) contents along the flow gradient was present in the seagrass, with 5-, 8- and 6-fold increases, respectively, but not in the saltmarsh (Fig. 2B–D, Table 1). The sediment contents of OM, OC and TN were, on average, higher in the saltmarsh (9.87 ± 1.57% OM, 2.25 ± 0.49% OC and 0.34 ± 0.07% TN) than in the seagrass habitat (5.93 ± 3.22% OM, 1.24 ± 0.85% OC and 0.17 ± 0.10% TN).

**Figure 3:**
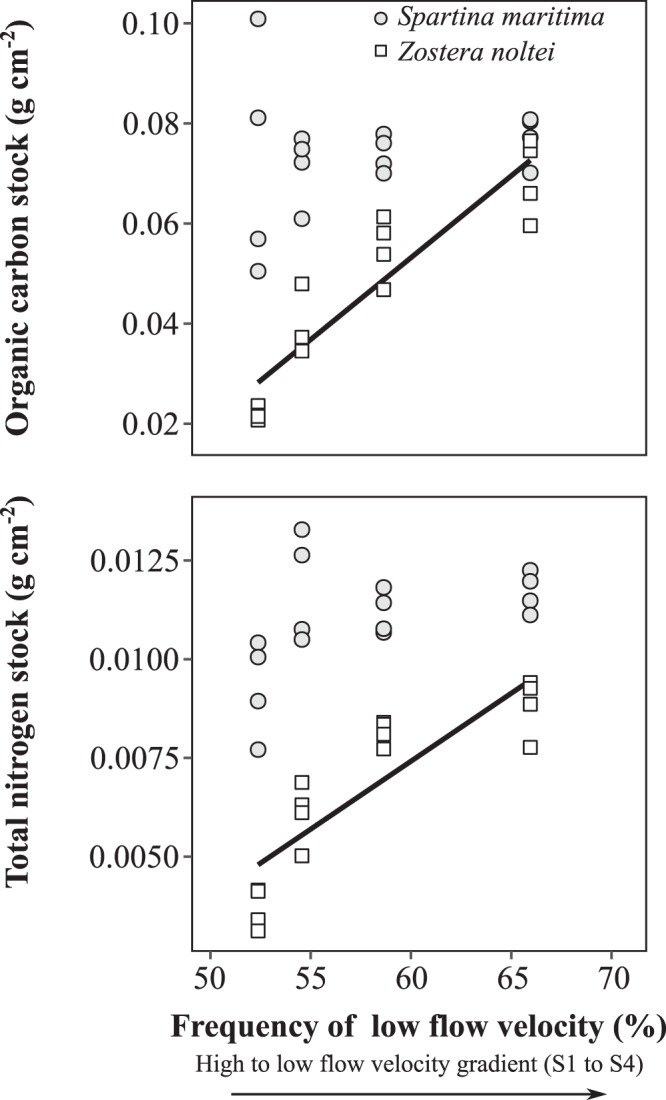
Relationships between *Spartina maritima* and *Zostera noltei* superficial sedimentary stocks of organic carbon (OC) and total nitrogen (TN), and the frequency of low flow velocities (≤0.2 m s^−1^) along sampling stations. Regression lines: OC - *Z. noltei* = – 0.1433 + 0.0033*x, R^2^ = 0.83, p < 0.001; TN *Z. noltei* = – 0.013240 + 0.00034*x, R^2^ = 0.72, p < 0.001. Linear regressions were not significant for *S. maritima*.

Superficial sedimentary OC stock in the seagrass increased 3-fold from S1 (0.022 ± 0.001 g OC cm-2) to S4 (0.069 ± 0.008 g OC cm-2), with an average of 0.046 ± 0.019 g OC cm-2 (Fig. 2E). In the saltmarsh, no differences were found in the OC stock along the flow gradient, being on average 0.073 g OC cm-2 (Fig. 2E). The sedimentary TN stock showed the same spatial variation as OC, with an averaged value of 0.011 ± 0.0014 g OC cm-2 in *S. maritima* sediments and 0.0067 ± 0.0021 g OC cm-2 in *Z. noltei* (Fig. 2F). The OC and TN stocks varied linearly along the flow gradient in the seagrass (OC: R^2^ = 0.83, p < 0.001); TN: R^2^ = 0.72, p < 0.001) but not in the saltmarsh (Fig. 3).

### Organic matter sources along the flow gradient

**Figure 4:**
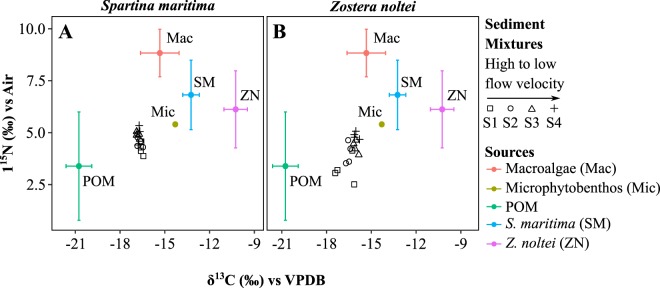
Isotopic signatures δ^15^N and δ^13^C of the sedimentary organic matter pool (mixtures) in *Spartina maritima* (**A**) and *Zostera noltei* (**B**) habitats from high to low current velocities, and signatures of organic matter sources in each habitat (mean ± standard deviation): POM - particulate organic matter, SM - *S. maritima*, ZN - *Z. noltei*, Mac – green macroalgae and Mic – microphytobenthos.

The δ^15^N and δ^13^C signatures of the sedimentary organic matter in *S. maritima* were similar among stations (Fig. 4A), indicating a lack of spatial variation in its organic matter sources. Contrastingly, the δ^15^N and δ^13^C signatures of *Z. noltei* sedimentary organic matter varied significantly (Fig. 4B), being the signatures in the most exposed station (S1) significantly lower than the signatures in the two most sheltered stations, S3 and S4. Separate mixing models were run for each station for both species, but the model results of *S. maritima* were pooled *a posteriori* due to the lack of spatial variability of sediment OM signatures, following published recommendations^41^. The range of stable isotope signatures of the sediment organic matter were within the ranges of the sources’ signatures, allowing to calculate the theoretical contribution of the sources to the sedimentary OM pool with the mixing model.

**Figure 5:**
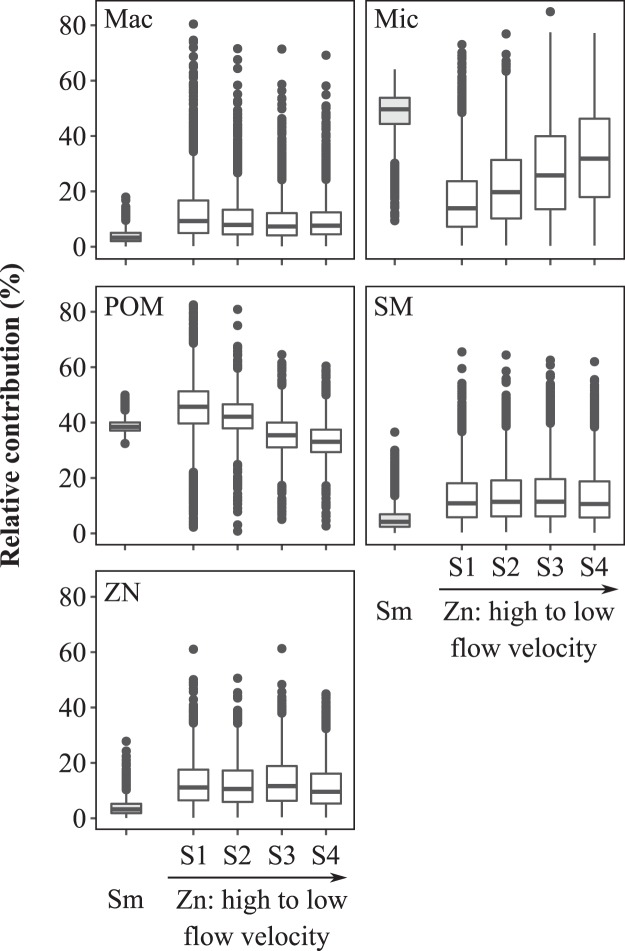
Theoretical contributions of organic matter sources (particulate organic matter, POM; *Spartina maritima*, SM; *Zostera noltei*, ZN; green macroalgae, Mac; microphytobenthos, Mic) estimated by the stable isotopes mixing model (SIMMR R package) in habitats of *S. maritima* (**A**) and *Z. noltei* (**B**). Contributions were combined in the analysis of *S. maritima* because no differences were found among the isotopic signatures of sedimentary organic matter along the sampling stations. The box-plot center line represents the median, hinges indicate the 25^th^ and 75^th^ quantiles, whiskers indicate 5^th^ and 95^th^ quantiles, and outliers are plotted as black dots.

The mixing model results revealed that within *Z. noltei*, POM and microphytobenthos were the main sedimentary OM sources, with the POM contribution decreasing along the flow gradient (45–35%) as opposed to the microphytobenthos, which contribution increased along the flow gradient (17–32%, Fig. 5). POM and microphytobenthos were also the main contributors to the sedimentary organic matter of *S. maritima* (48% and 39%, respectively)*. Z. noltei*, *S. maritima* and green macroalgae showed very low contributions (12% on average).

## Discussion

We report here, for the first time, a significant relationship between flow current velocity and OC and TN superficial sedimentary stocks in seagrasses, contrary to the saltmarsh where no relationship was found. The short-term stocks of organic carbon (OC) and total nitrogen (TN) in superficial lower intertidal sediments of the seagrass *Zostera noltei* increased ca. 3- and 2.4-fold, respectively, as flow velocity decreased. The predicted flow current velocities herein presented (Fig. 1) represent the unidirectional flow conveyance along the channel rather than the specific flow currents within the canopies of vegetation assemblages. Within canopies, the 3-dimensional physical structure and spatial heterogeneity lead to complex flow systems making difficult to characterize water flow. For unidirectional flow, the canopy drag may reduce the within-canopy velocity relative to that in adjacent open water by 70 to 90%^42^.

Seagrass meadows have been widely reported as globally important carbon stocks^3^, but only recently there have been an increasing number of studies investigating the habitat characteristics and mechanisms that influence their carbon sequestration (reviewed in^43^). Not all seagrass species and habitat characteristics have the same potential for carbon burial. Sediment traits such as dry density, porosity and silt/mud were identified as highly correlated with OC stocks, but only in small and fast-growing species such as *Zostera*, *Halodule* and *Halophila*^17,24,43,44^.

To the best of our knowledge, no attempts were reported relating seagrass OC stocks directly with flow current velocities, which ultimately determines the sediment deposition/resuspension rates and grain size, as well as the organic matter content^31^. Most studies show that sediments of seagrass habitats with predominantly lower energetic hydrodynamic conditions have higher OC content than of habitats with higher energetic hydrodynamic conditions. For example, the sheltered sites with low wave energy of the Mediterranean seagrass *Posidonia oceanica*, presented also higher carbon burial rates, but the fetch distance alone could not explain the differences among the meadows examined^45^. As well, the sediment OC content within northeastern Australian seagrasses was consistently higher in sites with lower wave height and higher turbidity^28^. Besides flow current velocities, the seagrass structural complexity and depth were also identified as important drivers for carbon stocks.

In contrast to low intertidal *Z. noltei*, the stocks of the higher intertidal *S. maritima* did not vary significantly along the flow gradient. However, the short-term OC and TN sequestration of *S. maritima* were 1.5- and 1.6-fold larger than within the seagrass, respectively, even though that habitat is located in a higher zone with reference to mean seal level and thus experiences shorter hydroperiods. Our observations also suggest that low flow current velocity is a main driver of high OC and TN stocks within the saltmarsh, because the tidal flow reaching the saltmarsh at the upper intertidal at the end of flood tides is highly reduced^46^ thus improving the settlement of fine grain sizes with high OC. In fact, the grain size within *S. maritima* was on average lower (and clay content higher) than within *Z. noltei* (Fig. 2), reflecting lower flow current velocity and therefore a larger potential to store organic matter. Flow velocities within saltmarshes are generally very low (e.g. < 1 cm s^−1^ ^47^) and there may be little or no net erosion, any losses being restored by the regular tidal inundation^48^. Saltmarsh flows of the order of 1 cm s^−1^ and higher flows within the seagrasses are consistent with the unidirectional flows of less than 20 cm s^−1^ along the channel, which were predicted here with the numerical model, reduced by 70–90% due to the canopy effect as reported in^42^.

The fact that *S. maritima* presented higher OC and TN stocks than *Z. noltei* in Ria Formosa lagoon, in spite of experiencing shorter hydroperiods, may be related not only with the local lower flow current velocities but also with differences in the canopy properties, which control how vegetation interacts with local hydrodynamical energy and consequently how particle trapping and reduced sediment resuspension is promoted^30,49^. Plant biomechanics (i.e. shoot flexibility) and meadow structure (i.e. shoot density) are two factors determining the extent of that interaction^50^. Stiff canopies such as those of *Spartina* species have a larger capacity to trap sediment than the flexible canopies of *Z. noltei*. Furthermore, the critical erosion shear stress for sediment re-suspension is higher in *S. maritima* than in *Z. noltei* surface sediments of Ria Formosa lagoon, due to higher contents of clay, Chl *a*, cyanobacteria, filamentous algae and colloidal carbohydrates^32^.

The observed increase of OC and TN with intertidal elevation is not a clear-cut, overall pattern^18^. The sediment accretion rates may be higher at lower intertidal zones^18,19^ whereas OC content may be higher at higher zones^51,52^. The reason for this discrepancy lies on the local trade-offs between hydroperiod, which decreases with elevation leading to lower sedimentation rates, and flow dynamics that decrease with elevation^46^ promoting the sedimentation of fine grain sizes with higher OC content.

The organic matter sources within the seagrass meadows, but not within the saltmarsh, varied along the flow gradient with increasing contributions of autochthonous microphytobenthos (17–32%) and decreasing contributions of allochthonous POM (45–35%). As well, sources varied along the intertidal vertical distribution as microphytobenthos contribution was highest at the higher intertidal saltmarsh (48%), but not POM (39%). This also suggests that flow current velocity is a major driver. The increased contribution of microphytobenthos to *Z. noltei* sediments with decreasing flow velocities and the higher contribution of microphytobenthos to the higher intertidal *S. marina* sediments, where flow velocities are lower, are probably related to lower re-suspension, a pattern that has been well described in tidal flats^53^. Important contributions of microphytobenthos and POM to sedimentary OC were also described elsewhere for seagrass meadows^54,55^. The decrease in the POM contribution along the channel and at higher intertidal levels is probably reflecting the trapping capacity of *Z. noltei* canopy^28,56^. Sediment trapping is an important ecological service of coastal vegetation as the decrease of suspended matter of the water column consequently increases the light penetration into the system, improving photosynthetic production.

The low contributions of *S. maritima* and *Z. noltei* as autochthonous sources of sedimentary organic matter results from the high contribution of seston POM, from the low belowground biomass production and from the export of leaves. The detached leaves of both species are carried elsewhere within the lagoon accumulating within the system^57^ or are exported to the adjacent coastal ocean through the inlets. Most of the floating leaves that are exported through the inlets are of *Z. nolteii* (non-published data). The leaves of this species are quite light, with a leaf mass area of 34.4 ± 7.4 g dw m^−2^, much below seagrass mean values (55.8 ± 25.7 g dw m^−2^)^58^, probably due to the high proportion of the aerenchyma they hold (about 60%^59^). These characteristics confer the leaves a high buoyancy, making them float away with the tidal flow. In meadows of seagrass species with heavy leaves, such as *Posidonia oceanica* (leaf mass area of 54.7 ± 8.4 g dw m^−2^ ^60^), the seagrass contribution to the sequestered OC was the most important source (43–94%^26^). The low contribution of autochthonous seagrass to the OC of seagrass sediment reported here (11–13%) is in contrast to the global estimate of about 50%^55^. This is probably reflecting the underrepresentation of small and fast-growing species such as *Z. noltei* on global estimates of blue carbon stocks and sources.

The average sedimentary OC content within the studied meadows of *S. maritima* and *Z. noltei* of Ria Formosa were 2.25 ± 0.49% and 1.24 ± 0.84%, respectively. The saltmarsh OC is low compared to the value of 5.40% reported in^13^, which does not discriminate the species composition, and it is still lower if compared to *S. alterniflora* in Florida and *S. anglica* in Denmark, which were both around 12%^38,61^. On the other hand, the *Z. noltei* OC content matches the average of 1.21 ± 1.19%, estimated from reports for *Zostera* species elsewhere^17,24,62–64^. The seagrass stocks reported here increase the global variability of seagrass carbon stocks^3^, which have been commonly biased for large and persisting species such as *Posidonia* spp^16^. As for surface sedimentary TN, the *Z. noltei* seagrass meadows of Ria Formosa presented one order of magnitude higher values (0.17 ± 0.10%) than those reported for its congeneric *Z. marina* in northern Spain (0.015 to 0.03%^36^), a site exposed to higher currents, where sediment is very sandy. The TN content in *S. maritima* sediments (0.34 ± 0.08%) was within the range of literature data (e.g. 0.04 to 0.79%^38^). Nitrogen sequestration is an important ecosystem service provided by coastal vegetated assemblages in Ria Formosa lagoon, particularly because this system receives high N inputs from waste water treatment works and groundwater^65^.

In conclusion, our findings show that flow current velocity is a key factor to consider in global estimates of short-term sedimentary carbon and nitrogen stocks of seagrasses, particularly in coastal systems with high tidal amplitudes and a complex system of channels, were important gradients of flow current velocities are established. Important differences were also found between higher intertidal saltmarsh and lower intertidal seagrasses, which highlight that local trade-offs between tidal currents, hydroperiod and structural complexity of vegetation must be considered, at both horizontal and vertical (elevation) spatial dimensions, for better estimates of blue carbon and nitrogen, and to better prioritize sites for conservation and restoration.

## Methods

### Site description

The Ria Formosa lagoon is a mesotidal system located in southern Portugal. The lagoon extends over 55 km along the coast, consists of two peninsulas and five islands, and it is connected to the ocean through six tidal inlets. Tides are semi-diurnal, with amplitudes ranging from 3.5 m on spring tides to 1.3 m on neap tides. The intertidal areas are mainly covered by the saltmarsh species *Spartina maritima* and the seagrass *Zostera noltei*, the latter occupying an estimated area of 1304 ha, which represent 45% of the total intertidal area^66^. Water circulation inside the lagoon is mostly driven by tides^67^. Due to the small freshwater inputs and the dominance of the tidal forcing on its circulation, the Ria Formosa is vertically well-mixed, with no evidence of haline or thermal stratification.

Four intertidal stations (S1 to S4, Fig. 1) were selected along a gradient of flow current velocity, from the main navigation channel closer to the main lagoon inlet to the inner part of a secondary channel. The flow gradient was predicted by applying a numerical model approach (see next section). Each station included two co-occurring habitats, *Z. noltei* (Zn) in the lower intertidal and *S. maritima* (Sm) in the upper intertidal.

### Hydrodynamic model

Depth-averaged current velocities were predicted for the sampling sites by applying a 2D modelling approach developed by Carrasco *et al*.^40^. The numerical model used is the Delft3D Flexible Mesh, a process-based unstructured grid finite volume model developed by Deltares. The mesh was developed by merging a curvilinear grid at the offshore, inlets and channels areas (with different resolutions) with triangular cells at the tidal flats and salt marsh areas of the lagoon, whereby triangulation is based on the Delaunay approach. The model domain of the mesh has a length in the alongshore direction that exceeds 30 km (western cell of the Ria Formosa lagoon), and it extends from Armona Inlet up to the western limit of Ancão Peninsula and reaches approximately 2 km offshore^40^.

The local bathymetry and topography were obtained from LIDAR data collected in 2011, which have a maximum resolution of 10 m, and from additional bathymetric surveys conducted over few shallow tidal channels. The model is forced with an offshore water level boundary and two lateral water level gradient boundary conditions (Neumann boundaries) with the main local tidal constituents. The model offshore boundary is close to the lagoon, and the tidal wave energy is absorbed by the lagoon system^40^. The amplitudes and phases of the constituents are derived from the TPXO global tidal model^68^.

The model was run for 60 days, following a spin-up time of 30 days, with a time step of 60 seconds. The model outputs consist of time-dependent water levels and depth-averaged velocities. Model calibration was carried out tuning the most appropriate bed roughness conditions and comparing the observed and predicted water levels (and tidal constituents) along the western sector of the Ria Formosa lagoon. For details on model calibration and validation see^40^. Depth-averaged currents were extracted from the calibrated model and used to characterize the velocity gradient in each sampling station (Fig. 1).

### Sediment sampling and analyses

The sediment sampling was conducted in November 2014 during spring low tide. Four replicated samples of superficial sediment (top 5 cm) of each habitat (Zn and Sm) at each station, were taken within the vegetation patches to avoid edge effects, using as corers plastic syringes with the bottom cut (diameter 2.5 cm, height 5 cm). For each replicate, eight sediment cores were pooled into plastic bags to reduce variability, then were transported to the laboratory in cool dark conditions and frozen (−20 °C) upon arrival for further processing. The wet volume of each sediment replicate was measured and then weighed before and after lyophilisation to determine dry bulk density (g dw cm^−3^). Further sediment analysis was made in sub-samples of each replicate, taken after homogenization. All samples, including the ones analysed for carbon and nitrogen content, were homogenized first by grounding manually in an agate mortar and then into Fritsh planetary Ball mill for 10 min.

Sediment grain size was determined after removing manually pieces of roots and leaves, removing salts by washing and removing organic matter with hydrogen peroxide attacks. Washing was done in a 250 ml glass cup with distilled water. The solution was then left for decantation during 24 hours after which most of supernatant was carefully withdrawn, and hydrogen peroxide added for organic matter attack. The fine particle size distribution (from 0 to 350 μm) was determined in the organic matter free fraction using a diffraction laser particle-size analyser (Mastersize 2000, Malvern Instruments Ltd.) after sediment resuspension in a dispersion agent (sodium polyphosphate). After being 1-mm sieved and homogenised in an agate ball mill, a subsample of the dry sediment was used to determine the percentage of organic matter (OM, % dw) by loss on ignition method (samples burnt at 450 °C for 4 h). Another subsample was used for elemental and isotopic analysis at the UH Hilo Analytical Laboratory (Hawaii, USA). Precision of isotopic analysis was 0.2‰. The organic carbon content (OC, % dw) and δ13C (vs Vienna Pee Dee Belemnite, ‰) of the organic fraction, were determined in the sediment samples after removal of the inorganic carbon fraction by acidification (1 M HCl), while total nitrogen (TN, % dw) and δ^15^N (vs air, ‰) were determined in untreated samples. Superficial sedimentary stocks of organic carbon and total nitrogen were calculated based on the initial dry bulk density, sampling depth (5 cm) and the % OC and % TN, respectively, and are reported as g m^−2^ in the top 5-cm of the sediment layer.

### Organic matter sources and stable isotopes mixing models

Five potential organic matter (OM) sources for the sedimentary OM pool were considered: *Spartina maritima* (SM), *Zostera noltei* (ZN), particulate organic matter suspended in the water column (POM), green macroalgae (Mac) and microphytobenthos (Mic). The epiphytes of *Z. noltei* leaves were not considered because their abundance is very low and their isotopic signature is within the variability of the signature of *Z. noltei*.

The relative contribution of the potential sources to the pool of surface sediment organic matter was investigated using Stable Isotope Bayesian mixing models (“simmr” R package version 0.3^69^). The models were run using the δ^13^C (from the organic fraction) and δ^15^N (from the total fraction) signatures of the sediment and the same signature of the 5 potential organic matter sources (SM, ZN and POM). The mean and standard deviations of isotopic signatures for those sources were obtained from values measured in samples collected in the Ria Formosa lagoon^70^ (and from R. Santos unpublished data): δ^13^C was –20.8 ± 0.9‰, n = 21 for POM, δ^13^C = –10.3 ± 0.8‰, n = 48 for ZN, δ^13^C = –13.2 ± 0.6‰, n = 18 for SM, δ^13^C = –15.3 ± 1.3‰, n = 12 for Mac and δ^13^C = –14.3‰, n = 1 for Mic; δ^15^N was 3.39 ± 2.61‰, n = 21 for POM, δ^15^N = 6.12 ± 1.86‰, n = 48 for ZN, δ^15^N = 6.82 ± 1.67‰, n = 18 for SM, δ^15^N = 8.8 ± 1.1‰, n = 12 for Mac and δ^15^N = 5.4‰ for Mic, n = 1. Only one sample was available for microphytobenthos. Spatial variability was included in the seagrass model but not in the saltmarsh model because in the seagrass the isotopic signatures of the sediment varied along the flow gradient (S1 to S4) whereas in the saltmarsh they did not. The isotopic signature of the sources was assumed to be constant among stations S1 to S4 and habitats, and concentration dependence was not incorporated into the models. Results of the mixing models are given as theoretical contribution (%) of each source to the sedimentary organic matter pool (mixtures).

### Statistical analysis

Data are presented as mean and standard deviation. Differences in sediment properties (mean grain size, organic matter, organic carbon, total nitrogen) among stations and habitats were examined using a 2-way analysis of variance (ANOVA) after checking model assumptions for normality and homoscedasticity (by visual inspections of the residual plots). Tukey’s pairwise comparisons were used to identify homogenous groups among stations and habitats when differences were found among them. Linear regression analysis was used to obtain relationships of sedimentary organic carbon and total nitrogen stocks with flow current velocity. A critical α level of 0.05 was used for all hypotheses tested. Data and statistical analyses were conducted in R programming software (R version 3.4.3).

## Data Availability

The datasets generated during and/or analysed during the current study are available from the corresponding author on reasonable request.
